# Ormosanine improves neuronal functions in spinal cord-injured rats by blocking peroxynitrite/calpain activity

**DOI:** 10.1515/tnsci-2020-0106

**Published:** 2020-05-29

**Authors:** Yan An, Jianing Li, Yajun Liu, Mingxing Fan

**Affiliations:** Beijing Jishuitan Hospital Search for articles by this author in MEDLINE®, Beijing, China; Department Of Spine Surgery, Beijing Jishuitan Hospital, Beijing 100035, China

**Keywords:** ormosanine, spinal cord injury, peroxynitrite, calpain, inflammation

## Abstract

The present study was performed to evaluate the effects of ormosanine against spinal cord injury (SCI) in rats and to examine the possible molecular mechanism of action. SCI was induced using an impactor device, and rats were treated with ormosanine 10, 50 or 100 mg/kg, p.o., for 10 days after induction of SCI. The effect of ormosanine on SCI was determined by estimating neurological functions and cytokines and parameters of oxidative stress level were estimated in SCI rats. Quantitative reverse transcription polymerase chain reaction, Western blotting analysis and histopathological study were performed on spinal tissue of SCI rats. The data suggested that treatment with ormosanine reversed the alterations of neurological function in SCI rats. Moreover, the levels of cytokines, oxidative stress and reactive oxygen species production were reduced in the ormosanine treatment group compared to the SCI group. The levels of calpain and neuronal nitric oxide synthase activity were significantly reduced in the spinal tissue of the ormosanine treatment group compared to the SCI group. Moreover, ormosanine treatment reduced the percentage of viable neurons in the spinal tissue of SCI rats. In conclusion, the results of this study showed that ormosanine treatment had a protective effect against neuronal injury in spinal cord-injured rats by regulating the peroxynitrite/calpain activity.

## 1 Introduction

Spinal cord injury (SCI) is a spinal cord damage that causes loss of function, such as feeling or mobility, due to increased production of several toxic metabolites [1]. The prevalence of SCI is increasing around the world, and it is a major health care issue with a significant socioeconomic impact [2]. SCI occurs due to ischemic, toxic and traumatic injury and further cellular damage or death is divided into two types, i.e., I^ry^ and II^ry^ SCI [3]. Several factors and pathways, i.e., excitotoxicity, redox and inflammatory mediators, are involved in the development of secondary injury leading to activation of neuronal apoptosis [4]. Activation of microglia stimulates the release of cytokines and reactive oxygen species (ROS), which contribute to the development of secondary injury in SCI [5]. Reduction of microglial activation ameliorates neuronal cell apoptosis, oxidative stress and inflammation in SCI [6]. Moreover, the bioavailability of nitric oxide (NO) is reduced in SCI, leading to overactivity of neuronal nitric oxide synthase (nNOS), which occurs due to excessive calcium overload [7]. This elevation of nNOS activity enhances the production of peroxynitrite, and activation of calpain occurs due to its overproduction, which contributes to the development of neuronal injury [8]. Inhibition of nNOS activity has a protective effect against neuronal injury. A number of drugs and surgical procedures are used for the management of SCI [9]. However, their effects are limited, and new drugs for the treatment of SCI are required.

Ormosanine is a pentacyclic alkaloid isolated from *Akebia quinata* (AQ) [10]. AQ, which is widely available in several parts of Asia, including Korea, Japan and China, has been reported to possess antibacterial, antioxidant, antidiuretic, anti-inflammatory and analgesic activities [11]. AQ has also been reported to contain several secondary metabolites such as chlorogenic acid, isochlorogenic acid A, isochlorogenic acid C, triterpenoid saponins and ormosanine, which has shown potential medicinal properties [12]. Ormosanine is one of the major chemical components of AQ, and it has also been reported to have antimalarial, analgesic, sedative, hypnotic and liver protective activities [13,14,15]. The present study was performed to evaluate the protective effects of ormosanine against secondary neuronal injury in SCI.

## 2 Materials and methods

### 2.1 Animals

Male Sprague–Dawley rats weighing 250–300 g were kept under a 12-h light/dark cycle at 60 ± 5% humidity and a temperature of 24 ± 3°C.


**Ethical approval:** The research related to animal use has been complied with all the relevant national regulations and institutional policies for the care and use of animals. The protocols used were approved by the Institutional Animal Care Committee of Beijing Jishuitan Hospital, China (IAEC/BJH/2018/03), and the animals were handled as per the Association for the Assessment and Accreditation of Laboratory Animal Care International (AAALAC) guidelines.

### 2.2 Chemicals

Ormosanine was provided by Kunming Institute of Botany, China. Enzyme-linked immunosorbent assay (ELISA) kits were purchased from R&D Systems (Minneapolis, MN, USA). Antibodies used in Western blotting analysis were obtained from Thermo Fisher Scientific (Wilmington, DE, USA).

### 2.3 Experimental

Following anaesthesia by intraperitoneal administration of 10 mg/kg xylazine and 90 mg/kg ketamine, the animals were placed on a stereotaxic apparatus. The spinal cord was exposed at the level of the thoracic vertebrae (T9–T10), and laminectomy was performed after making a dorsal median incision. SCI was induced as reported previously using a computer-operated impactor device with a contusion time of 85 ms, impact velocity of 1.5 m/s and tissue deformation of 2 mm. The incision was then closed using sutures. Fifty animals were divided into five different groups, with ten animals in each group: untreated control group (positive control group); SCI group (negative control group); and ormosanine 10, 50 and 100 mg/kg groups, which received ormosanine at doses of 10, 50 and 100 mg/kg, p.o., respectively, for 10 days.

### 2.4 Estimation of sensory function and motor behaviour

Locomotor activity was estimated by the open field test as reported previously [16]. Animals were allowed to move freely for 20 min in front of a light source, and the locomotor activity was estimated from the total number of circuit breaks. The Basso, Beattie and Bresnahan (BBB) score was estimated for all groups throughout the experimental period.

A *von Frey* plastic chamber was used to estimate mechanical allodynia as reported previously [17]. All animals were habituated to the test environment by placing them in the chamber for 2 h. An aesthesiometer was used to determine nociception by measuring the paw pressure threshold.

### 2.5 Estimation of cytokines

The serum levels of the inflammatory mediators, interleukin (IL)-1β, IL-6, nuclear factor kappa B (NF-κB) and tumour necrosis factor (TNF)-α, were determined by ELISA using commercial kits in accordance with the manufacturer’s protocols (R&D Systems).

### 2.6 Estimation of ROS

MitoSOX red mitochondrial superoxide indicator was used to estimate the levels of ROS in the intestinal tissues. Briefly, tissue homogenates were stained at 37°C in the dark for 30 min with 5 µM MitoSOX red. A fluorescent plate reader was used to estimate the intracellular ROS levels at excitation and emission wavelengths of 510 and 580 nm, respectively.

### 2.7 Determination of oxidative stress

Malondialdehyde (MDA) and superoxide dismutase (SOD) activities were estimated in intestinal tissues using ELISA kits according to the manufacturer’s instructions (R&D Systems).

### 2.8 Estimation of nitric oxide synthase activity

A nitric oxide synthase (NOS) assay kit was used to estimate the activity of NOS in the SCI tissues by determining the formation of l-[4,5-3*H*]citrulline from l-[4,5-3*H*] arginine. The total activity of constitutive NOS was estimated by subtracting the calcium-independent activity of NOS from the total NOS activity in pmol/mg protein/min.

### 2.9 Calpain activity assay

A calpain activity assay kit was used to estimate the activity of calpain in accordance with the manufacturer’s instructions. Each animal’s spinal tissue homogenate was incubated with reaction buffer and calpain substrate, and fluorometric analysis was performed to estimate cleavage of the substrate by calpain.

### 2.10 Quantitative reverse transcription polymerase chain reaction

Trizol reagent was used to isolate the total RNA from kidney tissues in accordance with the manufacturer’s instructions. A reverse transcription kit was used to reverse-transcribe cDNA from RNA in accordance with the manufacturer’s instructions. An ABI Prism 7500 system (Applied Biosystems, Foster City, CA, USA) was used with a SYBR green/fluorescein qPCR Master Mix kit (Thermo Fisher Scientific) with the following conditions: 50°C for 2 min; 95°C for 10 min; followed by 40 cycles at 95°C for 30 s and 60°C for 30 s. The resulting data were analysed using the comparative Ct method (2^−ΔΔCt^).

### 2.11 Primers (forward, reverse)

The primers used in this study are as follows: Caspase-3: 5′-GTGGAACTGACGATGATATGGC-3′, 5′-CGCAAAGTGACTGGATGAACC-3′; NF-κB: 5′-GAGCAAATGGTGAAGGAG-3′, 5′-TCTGGAAGTTGAGGAAGG-3′; Bax: 5′-CGGCGAATTGGAGATGAACTGG-3, 5′-CTAGCAAAGTAGAAGAGGGCAACC-3′; Bcl2: 5′-TGTGGATGACTGACTACCTGAACC-3′, 5′-CAGCCAGGAGAAATCAAACAGAGG-3′; and β-Actin: 5′-AGTGTGACGTTGACATCCGTAA-3′, 5′-GGACAGTGAGGCCAGGATAGA-3′.

### 2.12 Western blotting analysis

Western blotting analysis was performed to assess phospho-extracellular signal-regulated kinase (p-ERK), extracellular signal-regulated kinase (ERK), nNOS, phospho-nNOS (p-nNOS), calpain 1, calpain 2 and β-actin protein expression in the spinal cord tissue homogenate. A BCA assay kit (Thermo Fisher Scientific) was used to quantify the protein in the tissue homogenate, and 10% SDS-PAGE was performed to separate the proteins, which were electroblotted onto nitrocellulose membranes. Subsequently, the membranes were blocked with a blocking solution containing 5% non-fat milk and then incubated in blocking buffer with the following primary antibodies overnight at 4°C: p-ERK (1:100; Santa Cruz Biotechnology, Santa Cruz, CA, USA), ERK (1:100; Santa Cruz Biotechnology), nNOS (1:100; Santa Cruz Biotechnology), p-nNOS (1:100; Santa Cruz Biotechnology), calpain 1 (1:200; Cell Signaling Technology, Danvers, MA, USA), calpain 2 (1:200; Cell Signaling Technology) and β-actin (1:100; Santa Cruz Biotechnology). Later goat secondary antibody conjugated with horseradish peroxidase was added to the blocking buffer, and a chemiluminescence kit was used to detect the proteins.

### 2.13 Nissl staining

Each animal’s spinal cord tissues were isolated, dehydrated and embedded in paraffin. The isolated spinal cord tissue was cut into 10 µm thick sections using a microtome and stained with 1% cresyl violet. Image Pro Plus 6.0 software (Media Cybernetics, Silver Spring, MD, USA) was used to estimate the number of Nissl stain-positive neuronal cells.

### 2.14 Statistical analyses

All data are expressed as mean ± standard error of the mean (SEM; *n* = 10). Statistical analyses were performed by one-way analysis of variance. Post hoc comparisons were carried out with Dunnett’s test using GraphPad Prism software (ver. 6.1; GraphPad Software, Inc., San Diego, CA, USA). In all analyses, *P* < 0.05 was taken to indicate statistical significance.

## 3 Results

### 3.1 Ormosanine ameliorates the alterations in neurological function in SCI rats

**Figure 1:**
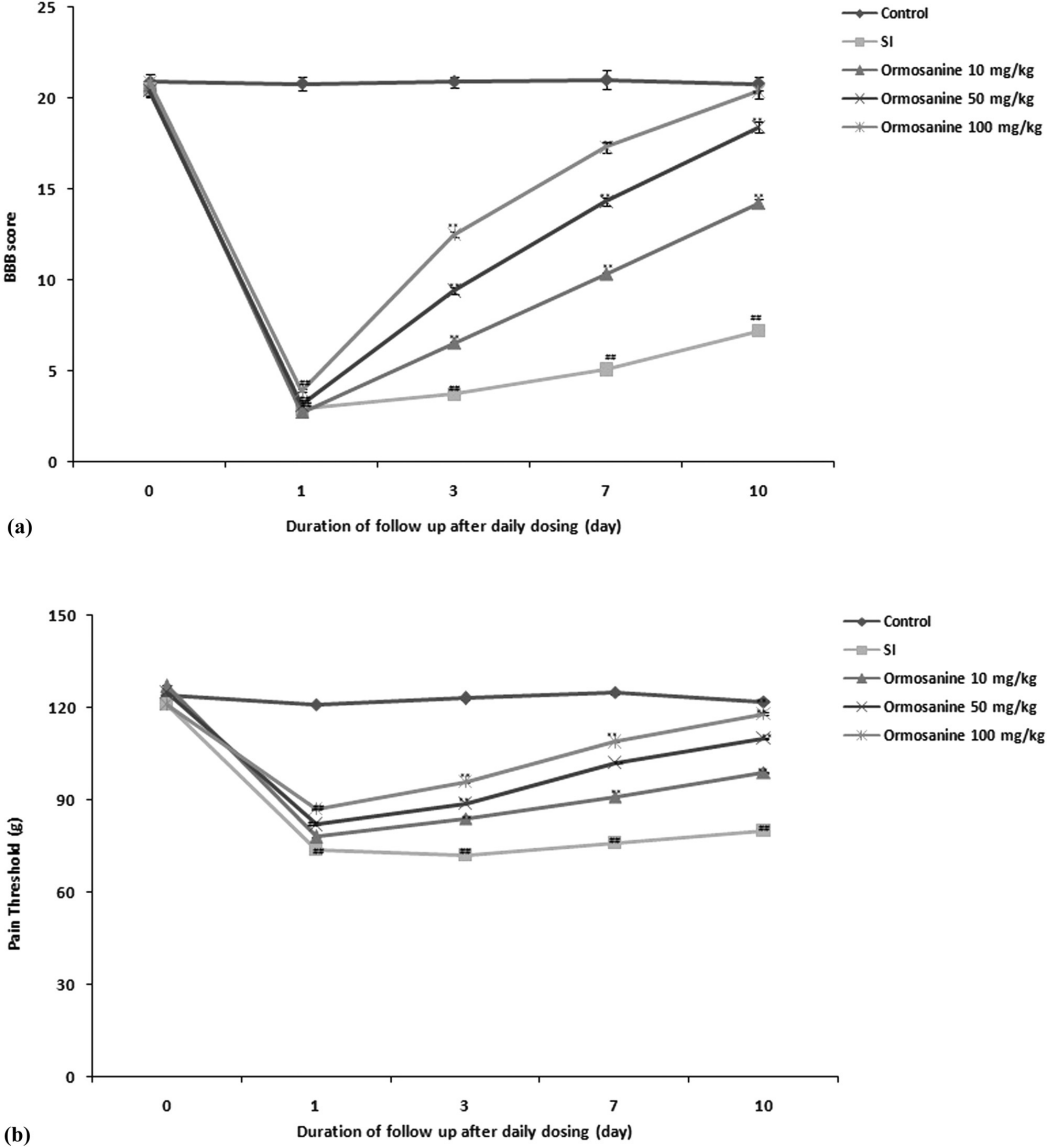
Ormosanine ameliorates the changes in neurological function scores in SCI rats. (a) Locomotor function estimated by the BBB score. (b) The pain threshold was examined to estimate neuropathic pain. Mean ± SEM (*n* = 10). ^##^ *P* < 0.01 vs control group; **P < 0.01 vs SCI group.

The effects of ormosanine on the neurological function scores were determined by examining locomotor function and pain threshold in SCI rats (Figure 1a and b). Locomotor function was examined based on the BBB score in each animal. The SCI group showed significantly reduced BBB scores compared to the control group on days 1, 3, 7 and 10 of the experimental period. However, treatment with ormosanine reversed the alteration in the BBB score in SCI rats in a dose-dependent manner (Figure 1a). Pain threshold was significantly reduced in the SCI group compared to the control group. Treatment with ormosanine improved the pain threshold in a dose-dependent manner compared to the SCI group throughout the experimental period (Figure 1b).

### 3.2 Ormosanine ameliorates the changes in ROS production in the spinal tissue of SCI rats

**Figure 2:**
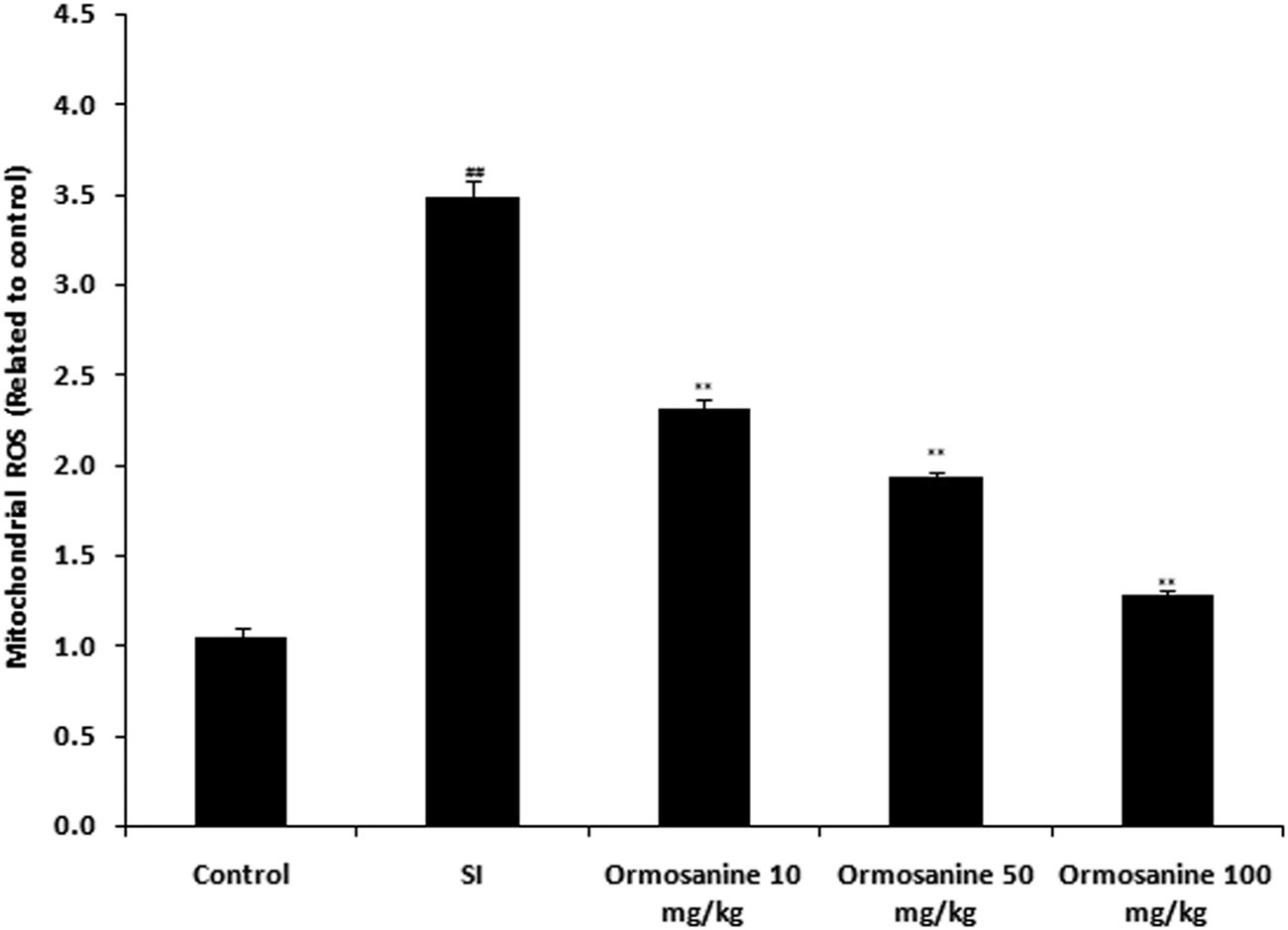
Ormosanine reduces the production of ROS in the spinal tissue of SCI rats. Mean ± SEM (*n* = 10). ^##^ *P* < 0.01 vs control group; **P < 0.01 vs SCI group.

Production of mitochondrial ROS was detected in the spinal cord tissue homogenate of ormosanine-treated SCI rats, as shown in Figure 2. Mitochondrial ROS production was significantly higher in the spinal tissue of the SCI group compared to that in the control group. The production of mitochondrial ROS was reduced by ormosanine treatment in a dose-dependent manner compared to the SCI group.

### 3.3 Ormosanine ameliorates the changes in inflammatory cytokine levels in the spinal tissue of SCI rats

**Figure 3:**
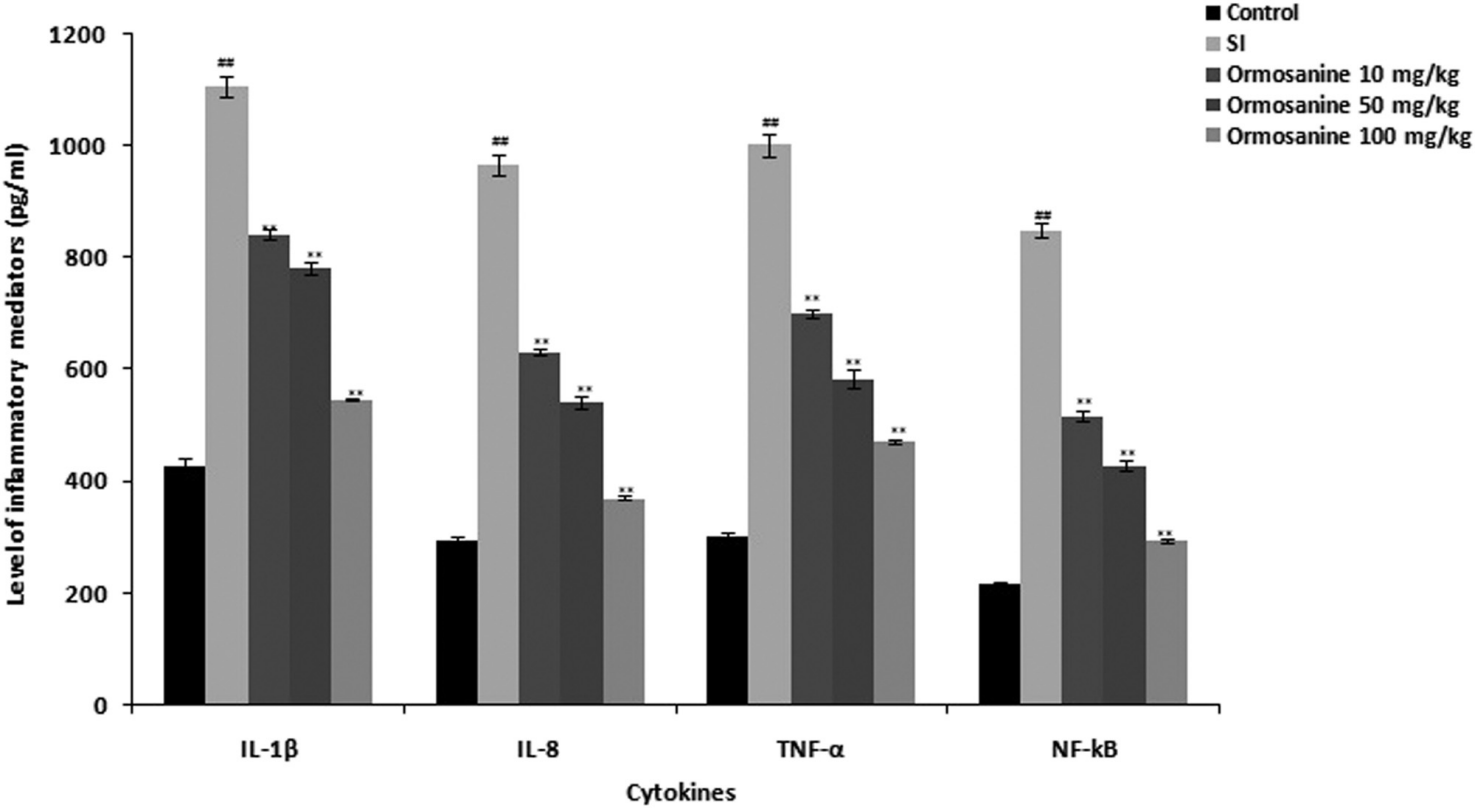
Ormosanine reduces inflammatory cytokine levels in the spinal tissue of SCI rats. Mean ± SEM (*n* = 10). ^##^ *P* < 0.01 vs control group; **P < 0.01 vs SCI group.

The levels of cytokines were estimated in the spinal tissue of ormosanine-treated SCI rats, as shown in Figure 3. The serum cytokine levels were increased in the SCI group compared to the control group. Treatment with ormosanine at different doses ameliorated these changes in serum cytokine levels in SCI rats in a dose-dependent manner.

### 3.4 Ormosanine ameliorates the changes in the parameters of oxidative stress in the spinal tissue of SCI rats

**Figure 4:**
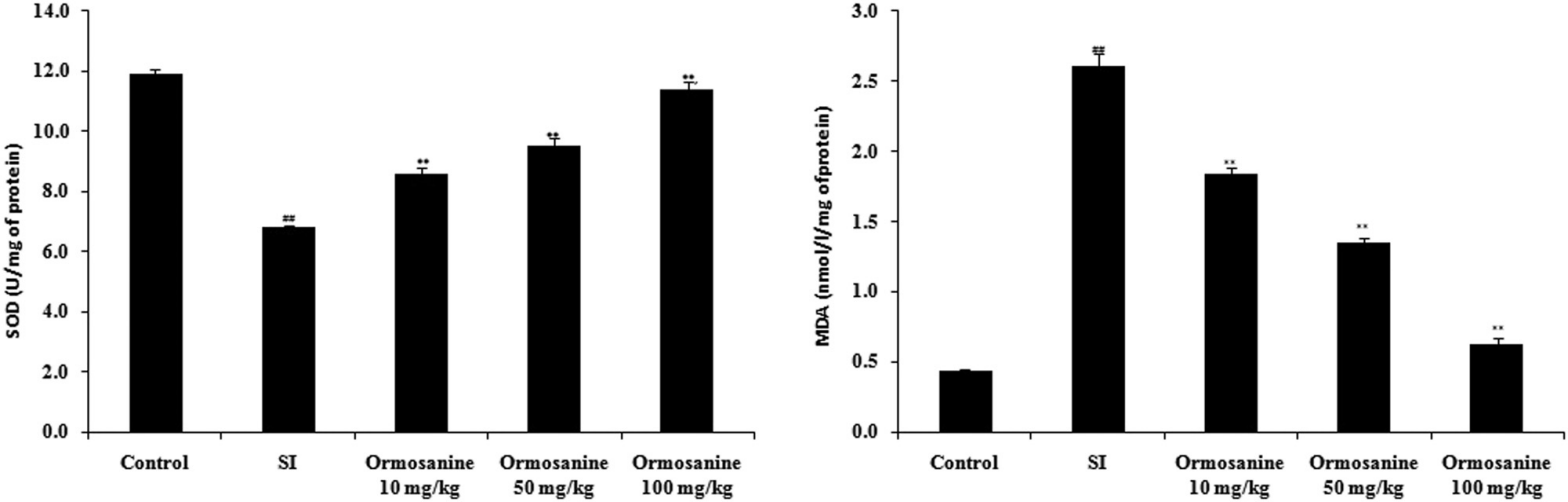
Ormosanine ameliorates the changes in parameters of oxidative stress in the spinal tissue of SCI rats. Mean ± SEM (*n* = 10). ^##^ *P* < 0.01 vs control group; **P < 0.01 vs SCI group.

Parameters of oxidative stress were determined in the spinal tissue of ormosanine-treated SCI rats, as shown in Figure 4. The SOD activity was reduced, whereas the MDA activity was increased in the spinal tissue of the SCI group compared to the control group. However, the ormosanine treatment groups showed increases in the SOD activity and reductions in the MDA activity in the spinal tissue compared to the SCI group.

### 3.5 Ormosanine ameliorates the changes in calpain and NOS activity in the spinal tissue of SCI rats

**Figure 5:**
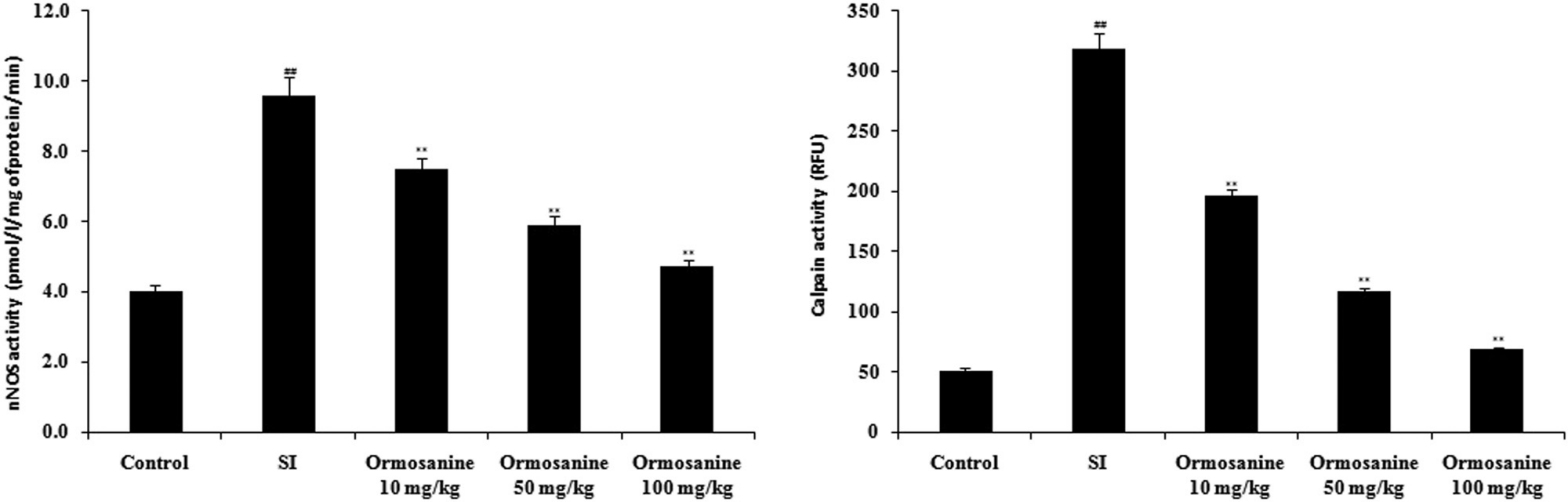
Ormosanine ameliorates the changes in activity of nNOS and calpain in the spinal tissue of SCI rats. Mean ± SEM (*n* = 10). ^##^ *P* < 0.01 vs control group; **P < 0.01 vs SCI group.

The effects of ormosanine treatment on the activities of calpain and nNOS in the spinal tissue of SCI rats were examined. The levels of calpain and nNOS activity were significantly increased in the spinal tissue of the SCI group compared to the control group. However, treatment with ormosanine at different doses ameliorated these changes in calpain and nNOS activity in the spinal tissue of SCI rats in a dose-dependent manner (Figure 5).

### 3.6 Ormosanine ameliorates the changes in NF-κB, Caspase-3, Bax and Bcl2 mRNA expression in the spinal tissue of SCI rats

**Figure 6:**
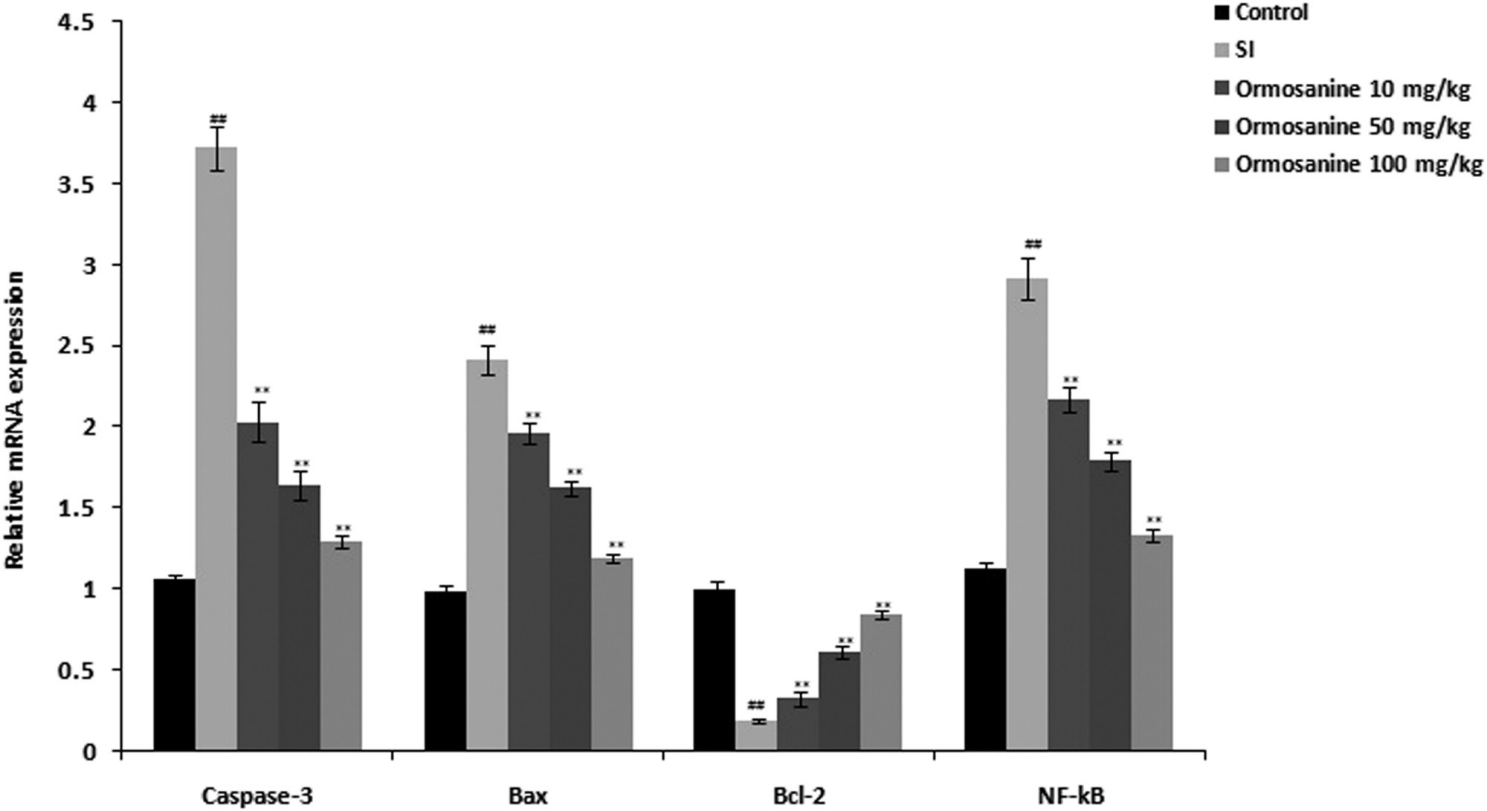
Effects of ormosanine on NF-κB, Caspase-3, Bax and Bcl2 mRNA expression in the spinal tissue of SCI rats. Mean ± SEM (*n* = 10). ^##^ *P* < 0.01 vs control group; **P < 0.01 vs SCI group.

Figure 6 shows the effects of ormosanine on NF-κB, Caspase-3, Bax and Bcl2 mRNA expression in the spinal tissue of SCI rats. The relative levels of NF-κB, Caspase-3 and Bax mRNA expression were significantly increased, and expression of Bcl-2 was significantly reduced in the spinal tissue of the SCI group compared to the control group. Treatment with ormosanine ameliorated the alterations in NF-κB, Caspase-3, Bax and Bcl2 mRNA expression in the spinal tissue of SCI rats.

### 3.7 Ormosanine ameliorates the changes in expression of nNOS, p-nNOS, calpain 1 and calpain 2 in the spinal tissue of SCI rats

**Figure 7:**
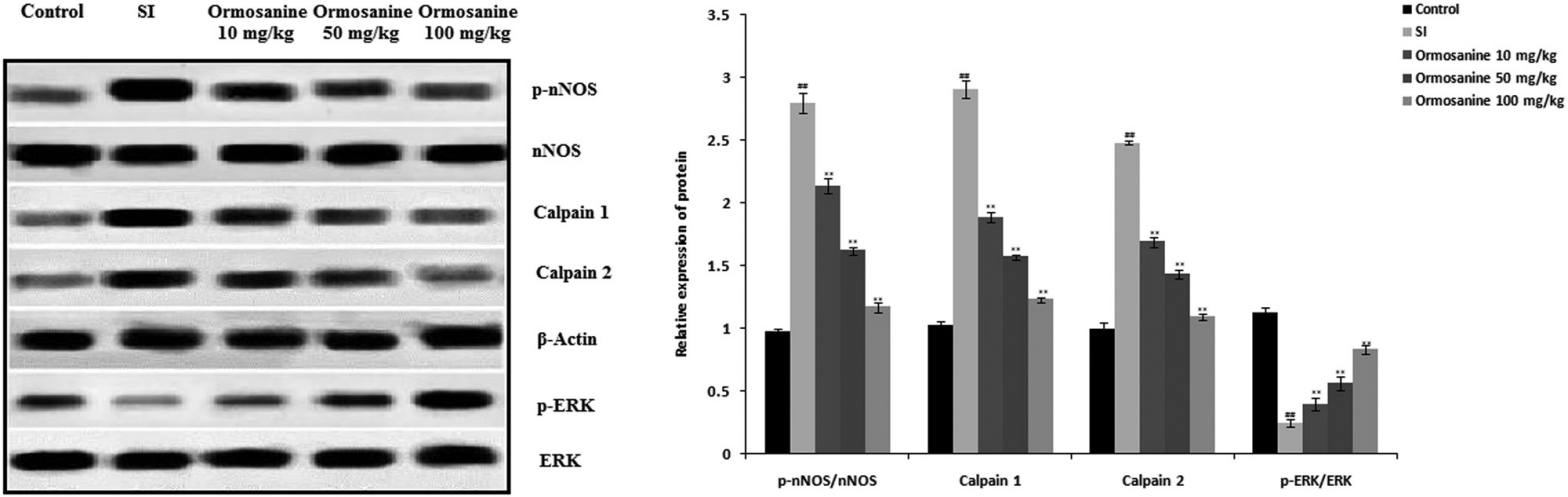
Effects of ormosanine on nNOS, ERK, calpain 1 and calpain 2 protein expression in the spinal tissue of SCI rats. Mean ± SEM (*n* = 10). ^##^ *P* < 0.01 vs control group; **P < 0.01 vs SCI group.

The levels of nNOS, calpain 1, calpain 2 and ERK protein expression were determined in the spinal tissue of ormosanine-treated SCI rats (Figure 7). The expression levels of nNOS, calpain 1 and calpain 2 protein were increased, and ERK protein expression was reduced in the spinal tissue of SCI rats. The changes in expression of nNOS, calpain 1, calpain 2 and ERK protein were ameliorated in the spinal tissue of the ormosanine treatment groups compared to the SCI group.

### 3.8 Ormosanine ameliorates the pathological changes in the spinal tissue of SCI rats

**Figure 8:**
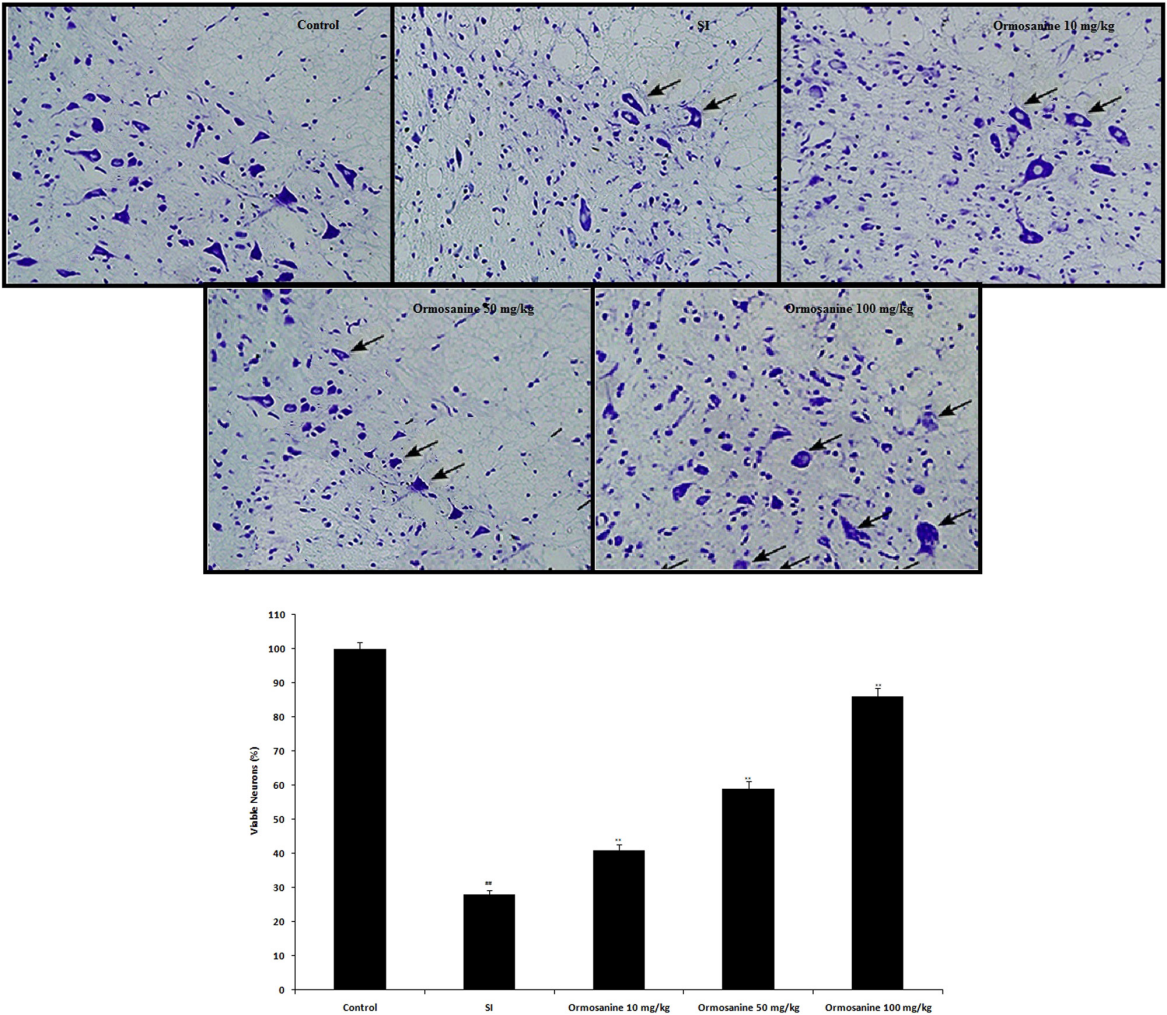
Effects of ormosanine on the percentage of viable neurons determined by Nissl staining in the spinal tissue of SCI rats. Mean ± SEM (*n* = 10). ^##^ *P* < 0.01 vs control group; **P < 0.01 vs SCI group.

Nissl staining was performed to estimate the percentage of viable neurons in the spinal tissue of SCI rats by histopathological analysis, as shown in Figure 8. The percentage of viable neurons was decreased in the SCI group compared to the control group. However, treatment with ormosanine ameliorated the change in the percentage of viable neurons in the spinal tissue of SCI rats.

## 4 Discussion

SCI is one of the major causes of permanent disability worldwide, including in China [18]. In SCI, neuronal injury occurs due to the progression of secondary injury, which involves several pathogenetic mechanisms [5], and novel treatments are required. The present study was performed to evaluate the effects of ormosanine in a rat SCI model by estimating neurological functions, such as pain threshold and locomotor function. The levels of cytokines, parameters of oxidative stress and production of ROS were examined in SCI rats. Moreover, calpain and NOS activities were determined, and quantitative reverse transcription polymerase chain reaction, Western blotting analysis and histopathological analysis were performed on the spinal tissue of SCI rats.

Secondary injury in SCI leads to activation of neuronal cell apoptosis due to enhanced release of inflammatory mediators, ischaemia, oedema and oxidative stress [19]. Primary injury in SCI enhances oxidative stress, which leads to increases in cytokine levels in the spinal tissue, resulting in the development of secondary injury [20]. Secondary injury in SCI occurs due to increased production of mitochondrial ROS, resulting in further secondary injury. The levels of cytokines, oxidative stress and ROS production were increased in the SCI group compared to the control group in the present study, and treatment with ormosanine ameliorated these effects. All of these alterations result in the progression of secondary neuronal injury, which further reduces the number of neurons by activation of neuronal apoptosis [21]. Ormosanine treatment was suggested to improve the number of viable neurons by reducing neuronal apoptosis, as it ameliorated the alterations in Caspase-3, Bax and Bcl2 mRNA expression in the spinal tissues of SCI rats. Caspase-3, Bax and Bcl2 proteins were reported to be involved in activation of the apoptosis pathway and were shown to stimulate apoptosis in SCI [22].

Activation of nNOS was reported to occur in the neuronal tissue following SCI, thus stimulating neuronal apoptosis; thus, inhibition of nNOS activity resulted in neuronal protection [23]. Moreover, nNOS-induced neuronal apoptosis in SCI causes neuronal degeneration by stimulating the activity of calpain [24]. Calpain inhibitor treatment or knockout was reported to prevent neuronal injury in SCI rats [25]. The findings of the present study supported these data and showed that treatment with ormosanine ameliorated the alterations in nNOS and calpain protein expression in the spinal tissue of SCI rats.

## 5 Conclusion

In conclusion, the results of the present study showed that ormosanine treatment protects against neuronal injury and reduces neuronal apoptosis in spinal cord-injured rats by regulating the peroxynitrite/calpain activity.
